# Supplementary material for: Piezo1 Mechano-Activation Is Augmented by Resveratrol and Differs between Colorectal Cancer Cells of Primary and Metastatic Origin
Source: Molecules. 2022 Aug 25;27(17):5430. doi: 10.3390/molecules27175430 (PMC9458129; doi:10.3390/molecules27175430)
Supplement: Supplementary file 1 [file molecules-27-05430-s001.zip › molecules-1832650-supplementary.pdf]

Supplementary Materials

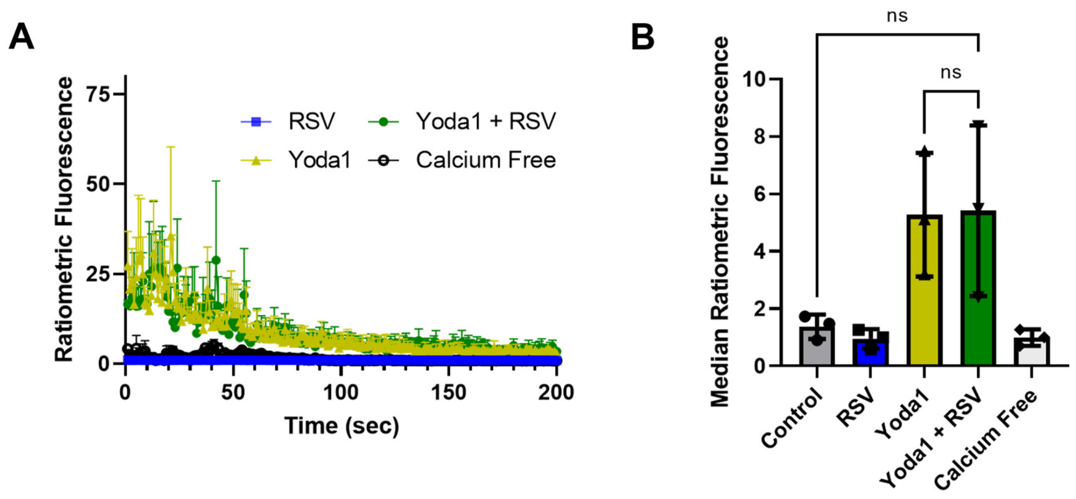

Figure S1. Resveratrol has no effect on calcium influx following Yoda1 treatment in SW620 cells. **(A)** Median ratiometric fluorescence as a function of time in SW620 cells. N=3. Error bars represent mean + SEM. **(B)** Average ratiometric calcium fluorescence over 200 sec after Yoda1 treatment. N=3, *ns* = not significant (one-way ANOVA with multiple comparisons). Error bars represent mean ± SD.

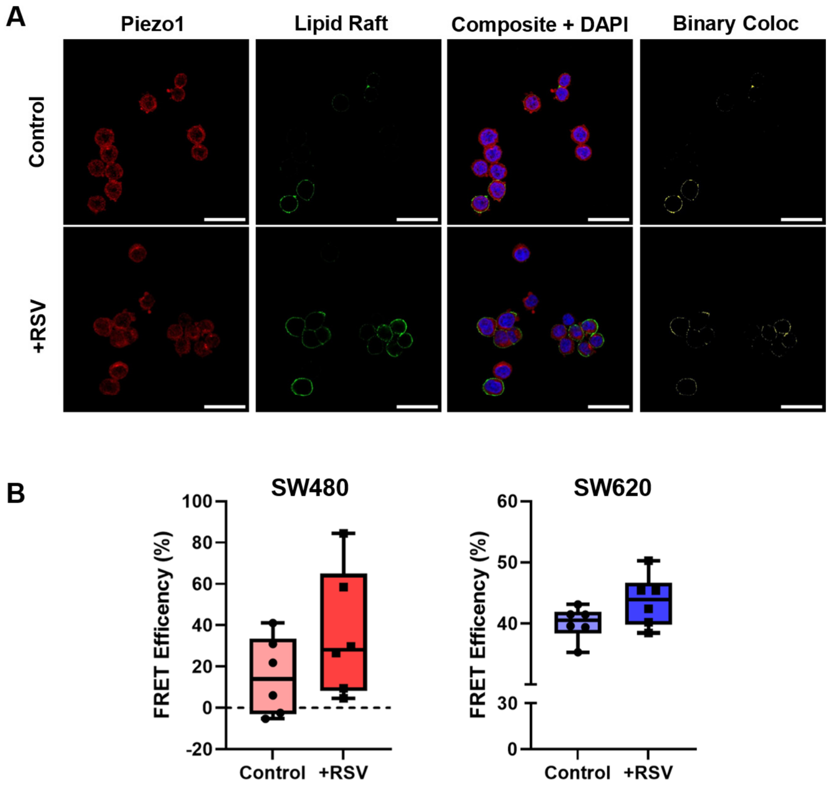

Figure S2. Resveratrol increases Piezo1-lipid raft colocalization. **(A)** Confocal microscopy images of Piezo1 (red), lipid rafts (green), DAPI (blue) and colocalization events between Piezo1 and LR (yellow) in SW620 cells. Scale bar = 30 μm. **(B)** FRET efficiency measured via flow cytometry for SW480 and SW620 cells. N=3 (n=6).

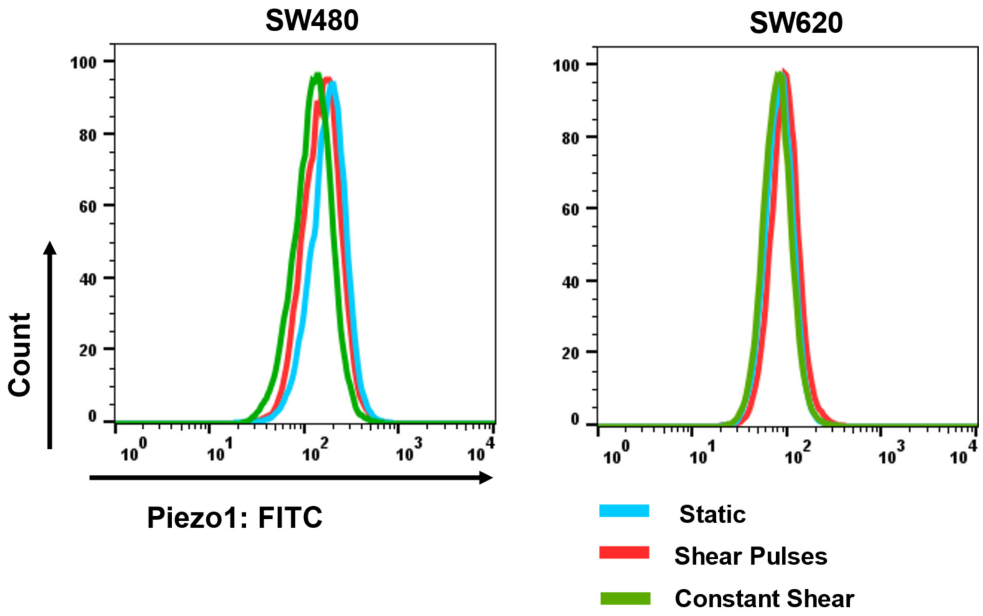

Figure S3. Fluid shear stress shows no appreciable effect on Piezo1 expression.

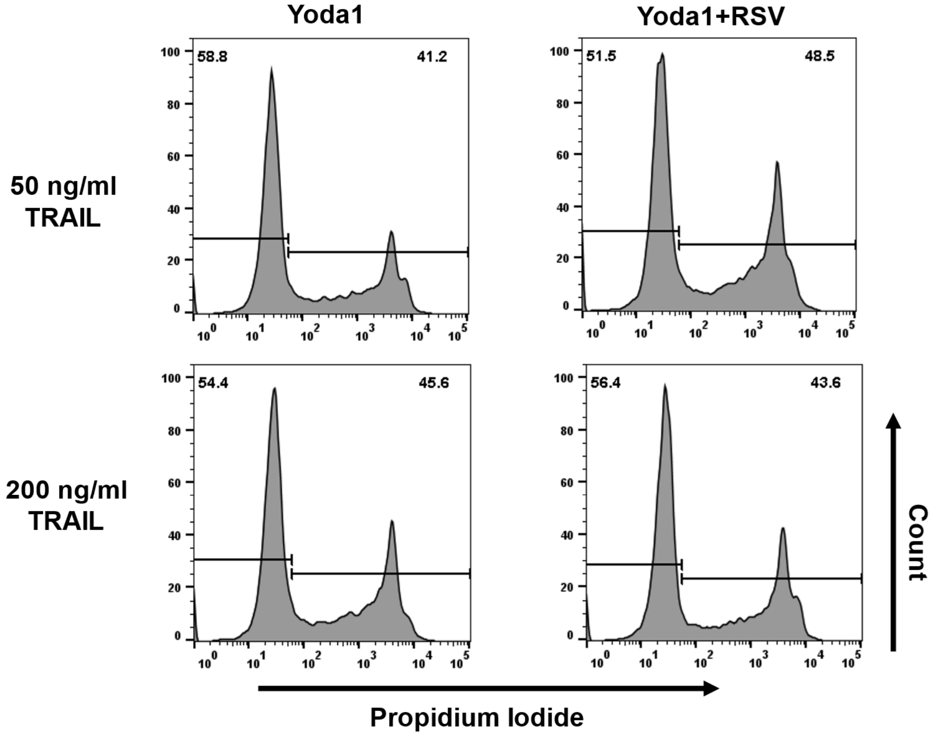

Figure S4. Cell viability is similar for SW480 cells treated with Yoda1 and 50 ng/ml or 200 ng/ml of TRAIL.

Table S1: List of reactions used in the computational model and their associated kinetic constants. (Adopted from Hope, J.M.; Lopez-Cavestany, M.; Wang, W.; Reinhart-King, C.A.; King, M.R. Activation of Piezo1 Sensitizes Cells to TRAIL-Mediated Apoptosis through Mitochondrial Outer Membrane Permeability. *Cell Death Dis.* **2019**, *10*, 837. https://doi.org/10.1038/s41419-019-2063-6.)

Reaction	k_i	k_{-i}	k_c	Ref.
$L + R \leftrightarrow L{:}R \rightarrow R^*$	$4 * 10^{-6}$	$5 * 10^{-3}$	$1 * 10^{-5}$	(57)
$flip + DISC \leftrightarrow flip{:}Disc$	$5 * 10^{-7}$	$5 * 10^{-4}$	NA	(57)
$pC8 + DISC \leftrightarrow pC8{:}DISC \rightarrow C8 + DISC$	$5 * 10^{-7}$	$5 * 10^{-4}$	1	(57)
$C8 + BAR \leftrightarrow C8{:}BAR$	$5 * 10^{-8}$	$5 * 10^{-4}$	NA	(57)
$pC3 + C8 \leftrightarrow pC3{:}C8 \rightarrow C3 + C8$	$5 * 10^{-8}$	$5 * 10^{-4}$	1	(57)
$pC6 + C3 \leftrightarrow pC6{:}C3 \rightarrow C6 + C3$	$5 * 10^{-7}$	$5 * 10^{-4}$	1	(57)
$pC8 + C6 \leftrightarrow pC8{:}C6 \rightarrow C8 + C6$	$6 * 10^{-9}$	$5 * 10^{-4}$	1	(57)
$XIAP + C3 \leftrightarrow XIAP{:}C3 \rightarrow XIAP + C3_{ub}$	$2 * 10^{-7}$	$1 * 10^{-4}$	$1 * 10^{-4}$	(57)
$PARP + C3 \leftrightarrow PARP{:}C3 \rightarrow cPARP + C3$	$1 * 10^{-7}$	$1 * 10^{-3}$	1	(57)
$Bid + C8 \leftrightarrow Bid{:}C8 \rightarrow tBid + C8$	$5 * 10^{-7}$	$1 * 10^{-4}$	1	(57)
$tBid + Bcl2c \leftrightarrow tBid{:}Bcl2c$	$1 * 10^{-8}$	$1 * 10^{-4}$	NA	(57)
$Bax + tBid \leftrightarrow Bax{:}tBid \rightarrow aBax + tBid$	$1 * 10^{-9}$	$1 * 10^{-4}$	1	(57)
$aBax \leftrightarrow MBax$	0.01	0.01	NA	(57)
$MBax + Bcl2 \leftrightarrow MBax{:}Bcl2$	$1 * 10^{-7}$	$1 * 10^{-4}$	NA	(57)
$MBax + MBax \leftrightarrow Bax2$	$1 * 10^{-7}$	$1 * 10^{-4}$	NA	(57)
$Bax2 + Bax2 \leftrightarrow Bax4$	$1 * 10^{-7}$	$1 * 10^{-4}$	NA	(57)
$Bax4 + Bcl2 \leftrightarrow Bax4{:}Bcl2$	$1 * 10^{-7}$	$1 * 10^{-4}$	NA	(57)
$Bax4 + Mito \leftrightarrow Bax4{:}Mito \rightarrow AMito$	$1 * 10^{-7}$	$1 * 10^{-4}$	1	(57)
$AMito + mCytoc \leftrightarrow AMito{:}mCytoc \rightarrow AMito + ACytoc$	$2 * 10^{-7}$	$1 * 10^{-4}$	10	(57)
$AMito + mSmac \leftrightarrow AMito{:}mSmac \rightarrow AMito + ASmac$	$2 * 10^{-7}$	$1 * 10^{-4}$	10	(57)
$ACytoc \leftrightarrow cCytoc$	0.01	0.01	NA	(57)
$APAF + cCytoc \leftrightarrow APAF{:}cCytoc \rightarrow APAF^*$	$5 * 10^{-8}$	$1 * 10^{-4}$	1	(57)
$APAF^* + pC9 \leftrightarrow Apop$	$5 * 10^{-9}$	$1 * 10^{-4}$	NA	(57)
$Apop + pC3 \leftrightarrow Apop{:}pC3 \rightarrow Apop + C3$	$5 * 10^{-8}$	$1 * 10^{-4}$	1	(57)
$ASmac \leftrightarrow cSmac$	0.01	0.01	NA	(57)
$Apop + XIAP \leftrightarrow Apop{:}XIAP$	$2 * 10^{-7}$	$1 * 10^{-4}$	NA	(57)
$cSmac + XIAP \leftrightarrow cSmac{:}XIAP$	$7 * 10^{-7}$	$1 * 10^{-4}$	NA	(57)
$calcium + calpain \leftrightarrow calpain{:}ca$	$5 * 10^{-10}$	$1 * 10^{-4}$	NA	(58)
$calcium + calpastatin \leftrightarrow calpastatin^*$	$5 * 10^{-10}$	$1 * 10^{-4}$	NA	NA
$calpastatin^* + calpain{:}ca \leftrightarrow calpastatin^* \rightarrow calpain_{bl}$	$3 * 10^{-7}$	$1 * 10^{-4}$	1	NA
$C3 + calpain_{bl} \leftrightarrow C3{:}calpain_{bl} \rightarrow C3 + calpain^*$	$1 * 10^{-7}$	$1 * 10^{-4}$	1	NA
$calpain^* + Bid \leftrightarrow calpain^*{:}Bid \rightarrow tBid + calpain^*$	$5 * 10^{-7}$	$1 * 10^{-4}$	1	NA
$calpain^* + Bcl2c \leftrightarrow calpain^*{:}Bcl2c \rightarrow calpain^* + cBcl2c$	$2 * 10^{-7}$	$1 * 10^{-4}$	0.1	NA

Table S2: Non-zero initial conditions utilized in the computational model.

Molecule	Basal initial condition (#/CC)	Ref.
$[\boldsymbol{Ligand}]$	$\mathbf{3000 = 50\frac{ng}{mL}}$<br>$\mathbf{0 = 0\frac{ng}{mL}}$<br>$\mathbf{30 = 0.05\frac{ng}{mL}}$<br>$\mathbf{12000 = 200\frac{ng}{mL}}$	(57)
$[\boldsymbol{Calcium}]$	$\boldsymbol{Calcium\ free\ buffer} = \mathbf{0.116}$ (μ$\boldsymbol{M}$)<br>$\boldsymbol{Control} = \mathbf{0.162}$ (μ$\boldsymbol{M}$)<br>$\boldsymbol{RSV} = \mathbf{0.134}$ (μ$\boldsymbol{M}$)<br>$\boldsymbol{Yoda1} = \mathbf{1}$ (μ$\boldsymbol{M}$)<br>$\boldsymbol{Yoda1 + RSV} = \mathbf{1.76}$ (μ$\boldsymbol{M}$)	*In vitro calcium values*
$[\boldsymbol{Receptor}]$	$\mathbf{200}$	(57)
$[\boldsymbol{FLIP}]$	$\mathbf{1 * 10^2}$	(57)
$[\boldsymbol{pC8}]$	$\mathbf{2 * 10^4}$	(57)
$[\boldsymbol{BAR}]$	$\mathbf{1 * 10^3}$	(57)
$[\boldsymbol{pC3}]$	$\mathbf{1 * 10^4}$	(57)
$[\boldsymbol{pC6}]$	$\mathbf{1 * 10^4}$	(57)
$[\boldsymbol{PARP}]$	$\mathbf{1 * 10^6}$	(57)
$[\boldsymbol{Bid}]$	$\mathbf{4 * 10^4}$	(57)
$[\boldsymbol{Bax}]$	$\mathbf{1 * 10^5}$	(57)
$[\boldsymbol{Mito}]$	$\mathbf{5 * 10^5}$	(57)
$[\boldsymbol{Cytochrome\ C}]$	$\mathbf{5 * 10^5}$	(57)
$[\boldsymbol{Smac}]$	$\mathbf{1 * 10^5}$	(57)
$[\boldsymbol{pC9}]$	$\mathbf{1 * 10^5}$	(57)
$[\boldsymbol{APAF}]$	$\mathbf{1 * 10^5}$	(57)
$[\boldsymbol{Calpain}]$	$\mathbf{1 * 10^5}$	(58)
$[\boldsymbol{Calpastatin}]$	$\mathbf{1 * 10^5}$	(NA)
$[\boldsymbol{cytosolic\ Bcl2}]$	$\mathbf{2 * 10^6}$	(57)
$[\boldsymbol{mitochondrial\ Bcl2}]$	$\mathbf{2 * 10^4}$	(57)

$[XIAP]$	$\mathbf{1 * 10^5}$	(57)

All species which are not listed have an initial condition equal to 1.
